# The secretome of stem cells isolated from the adipose tissue and Wharton jelly acts differently on central nervous system derived cell populations

**DOI:** 10.1186/scrt109

**Published:** 2012-05-02

**Authors:** Carlos A Ribeiro, Joana S Fraga, Mário Grãos, Nuno M Neves, Rui L Reis, Jeffrey M Gimble, Nuno Sousa, António J Salgado

**Affiliations:** 1 Life and Health Science Research Institute (ICVS), School of Health Sciences, University of Minho, 4710-057 Braga, Portugal; 2 Biocant - Center of Innovation in Biotechnology, 3060-197 Cantanhede, Portugal; 3 3B's Research Group - Biomaterials, Biodegradables and Biomimetics, University of Minho, Headquarters of the European Institute of Excellence on Tissue Engineering and Regenerative Medicine, AvePark, 4806-909 Caldas das Taipas, Guimarães, Portugal; 4 ICVS/3B's, PT Government Associate Laboratory, Braga/Guimarães, Portugal; 5 Stem Cell Laboratory, Pennington Biomedical Research Center, Louisiana State University System, Baton Rouge, LA 70808, USA

## Abstract

**Introduction:**

It is hypothesized that administration of stromal/stem cells isolated from the adipose tissue (ASCs) and umbilical cord (HUCPVCs) can ameliorate the injured central nervous system (CNS). It is still not clear, however, whether they have similar or opposite effects on primary cultures of neuronal populations. The objective of the present work was to determine if ASCs and HUCPVCs preferentially act, or not, on specific cell populations within the CNS.

**Methods:**

Primary cultures of hippocampal neurons were exposed to ASCs and HUCPVCs conditioned media (CM) (obtained 24, 48, 72 and 96 hours after three days of culture) for one week.

**Results:**

Cell viability experiments (MTS (3-(4, 5-dimethylthiazol-2-yl)-5-(3-carboxymethoxyphenyl)-2(4-sulfophenyl)-2H tetrazolium) test) revealed that CM obtained from both cell populations at all time points did not cause any deleterious effects on neuronal cells. In fact, it was determined that whenever the ASCs CM were supplemented with basic fibroblast growth factor (bFGF) and B27, there was a significant increase in the metabolic viability and neuronal cell density of the cultures. On the other hand, in the absence of CM supplementation, it was the HUCPVCs secretome that had the highest impact on the metabolic viability and cell density. In an attempt to unveil which factors could be involved in the observed effects, a screening for the presence of bFGF, nerve growth factor (NGF), stem cell factor (SCF), hepatocyte growth factors (HGF) and vascular endothelial growth factor (VEGF) in the CM was performed. Results revealed the presence of all these factors in ASCs CM, except bFGF; in contrast, in HUCPVCs CM it was only possible to detect robust NGF expression.

**Conclusions:**

Overall, the results confirm important differences on the secretome of ASCs and HUCPVCs, which lead to distinct effects on the metabolic viability and neuronal cell densities in primary cultures of hippocampal neurons; however, the factor(s) that promote the stronger effect of the HUCPVCs CM in neuronal survival is(are) still to be identified.

## Introduction

Currently there are no effective treatments for major central nervous system (CNS) injuries/disorders [1]. In the last decade, stem/progenitor cells isolated from the adipose tissue (ASCs) and the Wharton jelly of the umbilical cord have been proposed for possible transplantation as a therapy for CNS injuries [2-4]. Presently, it is commonly accepted that their potency is related mainly to their secretome, that is, to the production of molecules with a neuroregulatory character that support neuronal/glial cell survival and create an environment conducive to regeneration by endogenous cells [2,3].

Salgado *et al. *[5] demonstrated that the conditioned media (CM) of a population of mesenchymal progenitors isolated from the Wharton jelly, located in the perivascular region of the umbilical cord (human umbilical cord perivascular cells - HUCPVCs), were able to increase cell viability, survival and proliferation in primary cultures of hippocampal neurons and glial cells. Koh *et al. *[6] also revealed that the expression of granulocyte colony-stimulating factor (G-CSF), vascular endothelial growth factor (VEGF), glial derived neurotrophic factor (GDNF) and brain derived neurotrophic factor (BDNF) could be correlated with the neuroprotector effect revealed by stem cells isolated from the bulk of the Wharton jelly (WJ-MSCs), when transplanted to animal models of brain ischemia. Similar findings were also reported by Ding *et al. *[7] in an animal model of ischemic stroke. In this case the transplantation of human WJ-MSCs was not only able to promote functional recovery of behavioral deficits, but also the reduction of the lesion size, a higher extent of vascularization in ischemic areas and finally a higher expression of Stem Cell Derived Factor 1 (SDF-1), BDNF and GDNF in ischemic tissues. Identical outcomes were also observed in other animal models of injury within the CNS. For instance, Yang and colleagues [8] reported the improvement of spinal cord injured rats upon transplantation of undifferentiated WJ-MSCs and related these results with the expression of human neutrophil-activating protein-2 (NAP-2), neurotrophin-3 (NT-3), basic fibroblast growth factor (bFGF), glucocorticoid induced tumor necrosis factor receptor (GITR) and vascular endothelial growth factor receptor 3 (VEGFR-3). Finally, Weiss *et al. *[9] also revealed that WJ-MSCs could induce an overall improvement in the condition of an animal model of Parkinson's Disease (PD) through an increase of expression of GDNF at the site of injury.

Similar to what has been reported for stem cells isolated from the WJ's UC, growth factors such as VEGF, hepatocyte growth factor (HGF), bFGF, insulin like growth factor (IGF-1) and others have also been found in the ASCs secretome [10-12].

The ASCs application in models of injury, neurodegeneration and neurotoxicity is also well described. For instance Lee *et al. *[13] showed that ASCs transplantation into a mice model of Huntington Disease (HD) slowed down the disease progression by modulating the host pathogenesis. Lu and colleagues [14] also revealed that ASCs secretome exerted neuroprotection on glutamate mediated excitotoxicity in a PC12 cell line model. Moreover, this was partially related to the presence of different levels of BDNF, VEGF and HGF [15]. Another study using the PC12 cell line also reported interesting results [14]. In this particular work it was observed that ASCs conditioned media was able to induce cell neuritogenesis, and that this effect was partially mediated through a NGF related mechanism. Finally, ASCs application to a rat model of brain hypoxic-ischemic injury was also reported by Wei *et al. *[16]. Their objective was to study the role of concentrated conditioned media from cultured rat ASCs (ASC-CM) on the protection/recovery of the model. Both behavioral analysis and post-mortem evaluation of brain damage revealed that the conditioned media had a neuroprotective character. In this case IGF-1 and BDNF were indicated as the main mediators of the observed effects.

As it was shown, the effects of these cells on different CNS cell populations are relatively well described. However, a direct comparison of the effects of their secretome on CNS cells has not been performed. Therefore, the objective of the present work was to determine and compare the effects of the secretome of human ASCs and HUCPVCs on primary cultures of post-natal rat hippocampal neurons. For this purpose the latter were incubated with either conditioned media (CM) from ASCs or HUCPVCs. Results revealed that both ASCs and HUCPVCs secretome are able, in distinct forms, to increase cell viability and cell densities in the tested culture system, a fact that can be considered as an indicator of significant differences in their secretome composition.

## Methods

### Cell culture

#### Adipose tissue derived stem cells

ASCs were isolated according to a protocol previously described by Dubois *et al. *[17]. All protocols were reviewed and approved by the Pennington Biomedical Research Center Institutional Research Boards (IRB) prior to the study. Liposuction aspirates from subcutaneous adipose tissue sites (abdomen, flank, thighs) were obtained from female subjects undergoing elective plastic surgical procedures. All donors gave their written informed consent. Tissues were then digested in a 0.1% collagenase type I solution (Worthington Biochemical Corporation, Lakewood, NJ, USA) pre-warmed to 37°C for 60 minutes, after which they were centrifuged for five minutes at 300 g to 500 g at room temperature. The supernatant, containing mature adipocytes, was aspirated. The pellet was identified as the stromal vascular fraction (SVF). The SVF was resuspended and plated immediately in T225 flasks in Stromal Medium [DMEM/F 12 Ham's, 10% fetal bovine serum (Hyclone, Logan, UT, USA), 100 U penicillin/100 μg streptomycin/0.25 μg Fungizone ] at a density of 0.156 ml of tissue digest/cm^2 ^of surface area for expansion and culture. After reaching confluence, cells were passaged and kept in stromal medium.

#### Human umbilical cord perivascular cells

HUCPVCs were isolated from the UCs of local consenting full-term caesarean section patients. Ethical approval had been previously obtained from Hospital de S. Marcos, Braga, Portugal. Parents gave their written informed consent prior to the umbilical cord collection. Cells were isolated according to the procedure originally described by Sarugaser *et al. *[18]. Pieces of cord, 4 to 5 cm long, were dissected by first removing the epithelium of the UC section along its length to expose the underlying WJ. Each vessel, with its surrounding WJ matrix, was then pulled away and incubated in a 0.5 to 0.75 mg/ml collagenase (Sigma, St. Louis, MO, USA) with PBS (Gibco, Grand Island, NY, USA) solution for 18 hours. Supernatant was then diluted with PBS to reduce the viscosity of the suspension and centrifuged. Cells were further resuspended in culture media [α-MEM (Gibco) supplemented with 10% FBS (Gibco) and 1% antibiotic/antimycotic (Sigma)], plated in T75 flasks at a density of 4,000 cells/cm^2^. The culture medium was changed every two to three days. Upon confluence cells were trypsinized and passaged to new T75 flasks.

#### Hippocampal neurons

Hippocampal neuronal cultures were prepared from P4 Wistar rats [4]. Briefly, upon dissection, hippocampi were submitted to a trypsin based enzymatic digestion followed by mechanical dissociation. Isolated cells were then plated on Poly-D-Lysine (Sigma) coated coverslips at a density of 4,000 cells/cm^2^. Cultures were then incubated with CM and respective controls, as described in 2.2.

### Conditioned medium collection and experiments

CM was collected from P5 ASCs and HUCPVCs. For this purpose cells were plated out at a density of 4,000 cells/cm^2 ^and allowed to grow for three days. On day three, culture medium was renewed and CM were collected 24, 48, 72 and 96 hours thereafter and frozen. For CM collection Neurobasal-A medium supplemented with kanamycin (Gibco, 0.1 to mg/ml) was the chosen medium. Upon isolation, hippocampal neurons were plated out at the densities referred above and incubated from T0 with the previously collected and filtered CM (n = 3/CM time point) for seven days, after which cell viability and differentiation were assessed (see below). Two experimental setups were outlined: 1) B27 (Gibco) supplement and bFGF (Gibco, 10 ng/ml) were added to the CM prior to neuron incubation (control: standard Neurobasal-A media supplemented with the same concentrations of bFGF, B27 and kanamycin) and 2) CM were used as taken from the cultures flasks, without the addition of the supplements referred in 1).

### Cell viability assessment

Cell viability was assessed by the MTS test. The MTS (3-(4, 5-dimethylthiazol-2-yl)-5-(3-carboxymethoxyphenyl)-2(4-sulfophenyl)-2H tetrazolium) (Promega, Madison, WI, USA) test is an assay in which the substrate, MTS, is bioreduced into a brown formazan product by nicotinamide adenine dinucleotide phosphate (NADPH) or NADP produced by mitochondrial enzymes, which are active in living cells. Cell culture coverslips (n = 3) were placed in culture medium containing MTS in a 5:1 ratio and incubated in a humidified atmosphere at 37ºC and 5% CO_2_. After three hours of incubation, 100 μl of solution from each sample was transferred to 96 well plates and the optical density was determined at 490 nm. Results are shown as a ratio between CM and control incubated cultures.

### Immunocytochemistry

Cells were fixed in 4% paraformaldehyde for 30 minutes, permeabilized by incubation with 0.3% Triton X-100 in PBS for five minutes at room temperature (for neurons and astrocytes), and washed three times in PBS. Cells were then blocked with 10% FBS/PBS (60 minutes), followed by a 60-minute incubation with a mouse anti-rat microtubule associated protein 2 (MAP-2) (Sigma) antibody in order to detect mature hippocampal neurons. Cells were then washed in PBS and incubated with Alexa Fluor 594 goat anti-mouse immunoglobulin G (IgG). Primary antibody was omitted to produce negative controls. Samples were observed under an Olympus BX-61 Fluorescence Microscope (Olympus, Germany).

### Cell counts

Cell counts were performed by using Cell-P software (Olympus, Germany). For this purpose three cover slips per condition and three representative fields were chosen and analyzed. Results are shown as a ratio between the percentage of MAP-2 positive cells found in CM and control incubated cultures, respectively.

### Analysis of the conditioned media of ASCs and HUCPVCs

CM were assayed for cytokines using a Bio-plex human 5-plex panel immunoassay kit (Bio-Rad, Hercules, CA, USA), according to the manufacturer's instructions. The 5-plex panel consisted of the following analytes: bFGF, VEGF, NGF, SCF and HGF. A standard range of 0.2 to 3,200 pg/mL was used. CM samples were collected as previously described, centrifuged and frozen. Samples and controls were run in triplicate, standards and blanks in duplicate. Results were normalized to the total protein present in the respective CM.

### Statistical analysis

Statistical evaluation was performed using one way (hippocampal neurons experiments) analysis of variance (ANOVA) tests to assess the statistical differences. Statistical significance was defined as *P *< 0.05.

## Results and discussion

The present study set out to determine to what extent the secretome of two populations of human stromal/stem cells, ASCs and HUCPVCs, influence the viability and cellular densities of rat post-natal hippocampal neurons.

**Figure 1:**
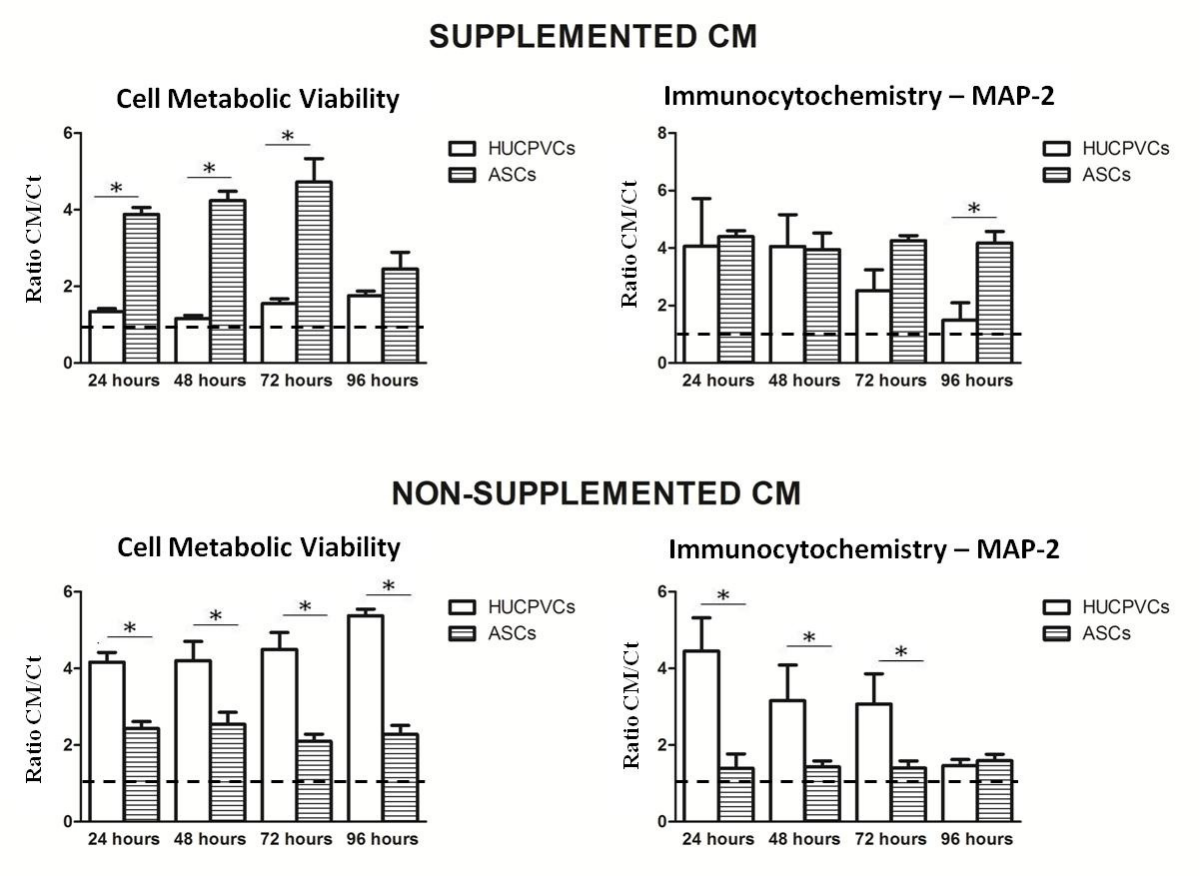
**Cell metabolic viability (MTS test) and cell densities, for MAP-2 positive cells, after incubation with ASCs and HUCPVCs supplemented and non-supplemented conditioned media (CM)**. Results for metabolic viability revealed that the action of ASCs CM was potentiated by the addition of exogenous bFGF and B27, leading to drastic increases in cell metabolic viability. On the other hand HUCPVCs CM did not need the addition of exogenous factors to induce similar effects in primary cultures of hippocampus. (Results shown as a ratio between CM and Control (Ct) n = 3 ± SD, one way ANOVA, *P *< 0.05). ANOVA, analysis of variance; ASCs, adipose tissue derived stem cells; bFGF, basic fibroblast growth factor; HUCPVCs, human umbilical cord perivascular cells; MTS, (4,5-dimethylthiazol-2-yl)-5-(3-carboxymethoxyphenyl)-2(4-sulfophenyl)-2H tetrazolium; SD, standard deviation.

**Figure 2:**
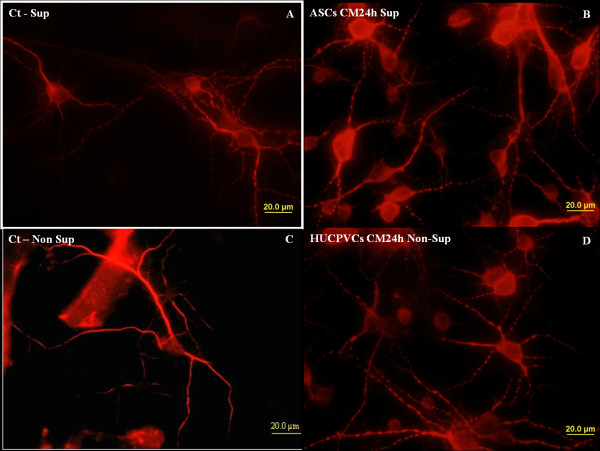
**Fluorescence microscopy micrographs of primary cultures of hippocampal neurons stained for the neuronal marker MAP-2, incubated under control conditions (A, C) and with ASCs supplemented CM (B) and non-supplemented HUCPVCs CM (D)**. As it can be observed both CM incubated cultures disclosed higher densities of MAP-2 positive cells, when compared to controls. ASCs, adipose tissue derived stem cells; CM, conditioned media; HUCPVCs, human umbilical cord perivascular cells; MAP-2, microtubule associated protein 2.

Figures 1 and 2 show the results obtained when hippocampal cultures were incubated for seven days with CM separately collected from ASCs or HUCPVCs. As it can be observed, none of the conditions used led to an overall decrease of cell metabolic viability among the primary hippocampal cultures in both supplemented and non-supplemented conditions (Figure 1). In supplemented CM the metabolic viability/activity of the neuronal cells was clearly upregulated in ASC CM (*P *< 0.05 for control and CM 24-72 h), even when compared to the results obtained for HUCPVCs CM. After this first and more general approach, an immunocytochemistry was performed for MAP-2 positive cells. This was performed, as the referred neuronal culture system is not 100% pure, presenting also astrocytes, neural progenitor and endothelial cells. The immunocytochemical analysis revealed that hippocampal cultures incubated with both ASCs and HUCPVCs supplemented CM had increased MAP-2 positive cell (neurons) densities when compared to control cultures. Still regarding this parameter, it was also possible to observe that for the 96-hour CM there were significant differences between ASCs and HUCPVCs CM (*P *< 0.05). Moreover, consistent with the MTS test, the secretome of the two stem cell populations displayed different temporal profiles. While cultures incubated with ASCs CM present similar values for MAP-2 positive cells throughout time (24-96 h), HUCPVCs CM presented a decaying temporal profile (Figure 1). However, when the conditions were changed and neuronal cells received the CM secretome alone (Figure 1), without the supplementation of bFGF and B27, the viability/metabolic assays results changed substantially. Under these conditions, the HUCPVCs CM-incubated cultures showed a clear up-regulation of the metabolic viability when compared to control and ASCs CM cultures (*P *< 0.05). Still, ASCs CM-incubated cultures also had a significant increase when compared to controls, but this was marginal when compared to that obtained with HUCPVCs CM. The determination of cell densities disclosed a similar trend (Figures 1 and 2). Overall, there was a decrease in the densities of MAP-2 positive cells for cultures incubated with non-supplemented CM, when compared with the supplemented CM conditions. The HUCPVCs secretome induced a clear positive response for the 24 h time point. There was a tendency to decay with time, as the HUCPVC 96-h CM values were slightly decreased when compared to those obtained for ASCs CM. These results, namely, the temporal profile disclosed by the HUCPVCs secretome are of note, as they are different from those reported by Salgado *et al. *[5] using P2 HUCPVCs. In that report HUCPVCs CM did not present a temporal profile regarding MAP-2 cell densities, a fact that may indicate that the secretome of these cells could be changing with the serial passaging of the cells.

In an attempt to determine which factors were being expressed by these cells, an antibody-based multianalyte Bio-Plex platform analysis (using the xMAP technology by Luminex™) for bFGF, NGF, SCF, HGF and VEGF, was conducted (Table 1). These growth factors were selected as they have been previously implicated in neuronal survival and differentiation [1,2]. It was possible to detect important variations between ASCs and HUCPVCs CM regarding the presence of these factors. While ASCs CM was positive for the presence of VEGF, NGF, SCF and HGF, HUCPVCs CM only revealed the presence of NGF and VEGF (and the latter just for one time point of collection). Thus, in a comparison between these two CM it becomes obvious that NGF is the only common factor present in similar amounts; moreover, both CM did not reveal the presence of bFGF. While the absence of bFGF in the ASCs CM seems to preclude the beneficial effects of this CM in neuronal survival and densities, it did not affect the actions of the HUCPVCs CM that might be promoted by NGF. However, these results also suggest that the differential effects of ASCs and HUCPVCs CM should not be attributed to any of these factors, as the HUCPVCs CM produced better results for cell survival and differentiation under no supplemented conditions, but rather to the presence of other, still unidentified, factors. Therefore, future work should be focused on the identification of such factors using more potent techniques such as those suggested by Zvonic [19] and She [20]. Additionally, it is also important to determine whether these differences would be maintained, if the CM used in the present report were injected into the rat hippocampus. Finally, it will also be important to compare the secretome of the cells under study, and its effects, to those of MSCs isolated from other sources, namely, bone marrow and embryonic stem cells [21-24].

**Table 1:** Quantification of bFGF, VEGF, NGF, SCF and HGF in ASCs and HUCPVCs CM (n = 3 + SD, results shown in pg/g of protein).

	bFGF	VEGF	NGF	SCF	HGF
ASCs CM24 h	0	50,174 ± 52,735	22,152 ± 1,768	1,028 ± 891	0
ASCS CM48 h	0	49,543 ± 39,746	1,872 ± 1,305	1,740 ± 2,699	1,626 ± 1,098
ASCs CM72 h	0	26,441 ± 16,484	1,800 ± 1,637	2,949 ± 2,546	4,752 ± 4,337
ASCs CM 96 h	0	27,984 ± 19,191	1,488 ± 835	2,311 ± 1,232	4,269 ± 5,036
HUCPVCs CM24 h	0	0	1,942 ± 1,325	0	0
HUCPVCs CM48 h	0	0	2,630 ± 1,520	0	0
HUCPVCs CM72 h	0	730 ± 537	2,139 ± 251	0	0
HUCPVCs CM96 h	0	0	1,311 ± 1,153	0	0

ASCs, adipose tissue derived stem cells; bFGF, basic fibroblast growth factor; HGF, hepatocyte growth factor; HUCPVCs, human umbilical cord perivascular cells; NGF, nerve growth factor; SCF, stem cell factor; SD, standard deviation; VEGF, vascular endothelial growth factor.

## Conclusions

The present work demonstrates that ASCs and HUCPVCs release trophic/neuroregulatory factors that improve the metabolic viability of hippocampal cultures. Importantly, it was possible to observe that their secretome acts differently on the cell viability and densities of post-natal cultures of hippocampal neurons. ASCs secretome effects are dependent on the interaction with added exogenous factors such as bFGF, while, on the other hand, HUCPVCs secretome is able to promote neuronal survival/differentiation in the absence of exogenous supplements.

## Abbreviations

ASCs: adipose tissue derived stem cells; BDNF: brain derived neurotrophic factor; bFGF: basic fibroblast growth factor; CM: conditioned media; CNS: central nervous system; FBS: fetal bovine serum; G-CSF: granulocyte colony-stimulating factor; GDNF: glial derived neurotrophic factor; HGF: hepatocyte growth factor; HD: Huntington Disease; HUCPVCs: human umbilical cord perivascular cells; IGF-1: insulin like growth factor 1; MAP-2: microtubule associated protein 2; MTS-3: (4,5-dimethylthiazol-2-yl)-5-(3-carboxymethoxyphenyl)-2(4-sulfophenyl)-2H tetrazolium; NAP-2: neutrophil-activating protein 2; NGF: nerve growth factor; NT-3: neurotrophin 3; SCF: stem cell factor; SDF-1: stem cell derived factor 1; SVF: stromal vascular fraction; UC: umbilical cord; VEGF: vascular endothelial growth factor; VEGFR-3: vascular endothelial growth factor receptor 3; WJ-MSCs: Wharton jelly mesenchymal stem cells.

## Competing interests

The authors declare that they have no competing interests.

## Authors' contributions

CAR conceived and executed all the experiments, interpreted and analyzed the data and drafted the manuscript. JSF was responsible for cell culture experiments, collection and assembly of data. MG executed the Bioplex assays and consequent data analysis and interpretation. NMN and RLR were involved in the conception of the experiments. JMG isolated and characterized the ASCs and revised the manuscript. NS conceived the experiments, analyzed and interpreted the data, and revised the manuscript. AJS conceived all the experiments, analyzed and interpreted the data, drafted the manuscript and revised the manuscript. All authors read and approved the final manuscript.

## References

[B1] Parr AM Tator CH Keating A Bone marrow derived mesenchymal stromal cells for the repair of central nervous system injury Bone Marrow Transp 2007 40 609 619 10.1038/sj.bmt.1705757 17603514

[B2] Salgado AJ Reis RL Sousa NJ Gimble JM Adipose tissue derived stem cells secretome: soluble factors and their roles in regenerative medicine Curr Stem Cell Res Ther 2010 5 103 110 10.2174/157488810791268564 19941460

[B3] Carvalho MM Teixeira FG Reis RL Sousa N Salgado AJ Mesenchymal stem cells in the umbilical cord: phenotypic characterization, secretome and applications in central nervous system regenerative medicine Curr Stem Cell Res Ther 2011 6 221 228 10.2174/157488811796575332 21476975

[B4] Cun-Gang F Qing-jun Z Jing-ru Z Therapeutic potentials of mesenchymal stem cells derived from human umbilical cord Stem Cell Rev 2011 7 195 207 10.1007/s12015-010-9168-8 20676943

[B5] Salgado AJ Fraga JS Mesquita AR Neves NM Reis RL Sousa N Role of human umbilical cord mesenchymal progenitors conditioned media in neuronal/glial cell densities, viability, and proliferation Stem Cells Dev 2010 19 1067 1074 10.1089/scd.2009.0279 19705968

[B6] Koh SH Kim KS Choi MR Jung KH Park KS Chai YG Roh W Hwang SJ Ko HJ Huh YM Kim HT Kim SH Implantation of human umbilical cord-derived mesenchymal stem cells as a neuroprotective therapy for ischemic stroke in rats Brain Res 2008 1229 233 248 18634757 10.1016/j.brainres.2008.06.087

[B7] Ding DC Shyu WC Chiang MF Lin SZ Chang YC Wang HJ Su CY Li H Enhancement of neuroplasticity through upregulation of beta1-integrin in human umbilical cord-derived stromal cell implanted stroke model Neurobiol Dis 2007 27 339 353 10.1016/j.nbd.2007.06.010 17651977

[B8] Yang CC Shih YH Ko MH Hsu SY Cheng H Fu YS Transplantation of human umbilical mesenchymal stem cells from Wharton's jelly after complete transection of the rat spinal cord PLoS One 2008 3 e3336 10.1371/journal.pone.0003336 18852872 PMC2566594

[B9] Weiss ML Medicetty S Bledsoe AR Rachakatla RS Choi M Merchav S Luo Y Rao MS Velagaleti G Troyer D Human umbilical cord matrix stem cells: preliminary characterization and effect of transplantation in a rodent model of Parkinson's disease Stem Cells 2006 24 781 792 10.1634/stemcells.2005-0330 16223852

[B10] Rehman J Traktuev D Li J Merfeld-Clauss S Temm-Grove CJ Bovenkerk JE Pell CL Johnstone BH Considine RV March KL Secretion of angiogenic and antiapoptotic factors by human adipose stromal cells Circulation 2004 109 1292 1298 10.1161/01.CIR.0000121425.42966.F1 14993122

[B11] Wang M Crisostomo PR Herring C Meldrum KK Meldrum DR Human progenitor cells from bone marrow or adipose tissue produce VEGF, HGF, and IGF-I in response to TNF by a p38 MAPK-dependent mechanism Am J Physiol Regul Integr Comp Physiol 2006 291 R880 884 10.1152/ajpregu.00280.2006 16728464

[B12] Nakagami H Maeda K Morishita R Iguchi S Nishikawa T Takami Y Kikuchi Y Salto Y Tamai K Ogihara T Kaneda Y Novel autologous cell therapy in ischemic limb disease through growth factor secretion by cultured adipose tissue-derived stromal cells Arterioscler Thromb Vasc Biol 2005 25 2542 2547 10.1161/01.ATV.0000190701.92007.6d 16224047

[B13] Lee ST Chu K Jung KH Im WS Park JE Lim HC Won CH Shin SH Lee SK Kim M Roh JK Slowed progression in models of Huntington disease by adipose tissue stem cells transplantation Ann Neurol 2009 66 671 681 19938161 10.1002/ana.21788

[B14] Lu S Lu C Han Q Li J Liao L Zhao RC Adipose-derived mesenchymal stem cells protect PC12 cells from glutamate excitotoxicity-induced apoptosis by upregulation of XIAP through PI3-K/Akt activation Toxicology 2011 279 189 195 10.1016/j.tox.2010.10.011 21040751

[B15] Tan B Luan Z Wei X He G Wei G Johnstone BH Farlow M Du Y AMP-activated kinase mediates adipose tissue stem cell-stimulated neuritogenesis of PC12 cells Neuroscience 2011 181 40 47 21352901 10.1016/j.neuroscience.2011.02.038

[B16] Wei X Du Z Zhao L Feng D Wei G He Y Tan J Lee WH Hampel H Dodel R Johnstone BH March KL Farlow MR Du Y The conditioned media of adipose stromal cells protect against hypoxia-ischemia-induced brain damage in neonatal rats Stem Cells 2009 27 478 488 10.1634/stemcells.2008-0333 19023032

[B17] Dubois SG Floyd EZ Zvonic S Kilroy G Wu X Carling S Halvorsen YD Ravussin E Gimble JM Isolation of human adipose-derived stem cells from biopsies and liposuction specimens Method Mol Biol 2008 449 69 79 10.1007/978-1-60327-169-1_5 18370084

[B18] Sarugaser R Lickorish D Baksh D Hosseini MM Davies JE Human umbilical cord perivascular cells (HUCPV) cells: a source of mesenchymal progenitors Stem Cells 2005 23 220 229 10.1634/stemcells.2004-0166 15671145

[B19] Zvonic S Lefevre M Kilroy G Floyd ZE DeLany JP Kheterpal I Gravois A Dow R White A Wu X Gimble JM Secretome of primary cultures of human adipose-derived stem cells: modulation of serpins by adipogenesis Mol Cell Proteomics 2007 6 18 28 17018519 10.1074/mcp.M600217-MCP200

[B20] She YM Rosu-Myles M Walrond L Cyr TD Quantification of protein isoforms in mesenchymal stem cells by reductive dimethylation of lysines in intact proteins Proteomics 2012 12 369 379 10.1002/pmic.201100308 22144135 PMC3440571

[B21] Ramos-Mejia V Bueno C Roldan M Sanchez L Ligero G Menendez P Martin M The adaptation of human embryonic stem cells to different feeder-free culture conditions is accompanied by a mitochondrial response Stem Cells Dev 2012 21 1145 1155 10.1089/scd.2011.0248 21671728

[B22] Ramos-Mejía V Fernández AF Ayllón V Real PJ Bueno C Anderson P Martín F Fraga MF Menendez P Maintenance of human embryonic stem cells in mesenchymal stem cell-conditioned media augments hematopoietic specification Stem Cells & Dev 2011 in press 10.1089/scd.2011.0400 21936705

[B23] Sánchez L Gutierrez-Aranda I Ligero G Rubio R Muñoz-López M García-Pérez JL Ramos V Real PJ Bueno C Rodríguez R Delgado M Menendez P Enrichment of human ESC-derived multipotent mesenchymal stem cells with immunosuppressive and anti-inflammatory properties capable to protect against experimental inflammatory bowel disease Stem Cells 2011 29 251 262 10.1002/stem.569 21732483

[B24] Sánchez L Gutierrez-Aranda I Ligero G Martín M Ayllón V Real PJ Ramos-Mejía V Bueno C Menendez P Maintenance of human embryonic stem cells in media conditioned by human mesenchymal stem cells obviates the requirement of exogenous basic fibroblast growth factor supplementation Tissue Eng Part C Methods 2012 18 387 396 10.1089/ten.tec.2011.0546 22136131

